# Editorial

**DOI:** 10.1007/s12286-020-00471-4

**Published:** 2020-12-09

**Authors:** Hans-Joachim Lauth, Martha Suda, Christoph Mohamad-Klotzbach

**Affiliations:** Julius-Maximilians-Universität Würzburg, Würzburg, Germany

A challenging but eventful year draws to a close: The global COVID-19 pandemic has also left its mark on the Journal of Comparative Politics (ZfVP)—especially during the first half of the year. We are therefore all the more pleased that our four multifaceted issues published in 2020 have received great interest. One area where this is visible is in the number of steadily increasing downloads. In September 2020 we recorded over 55,000 downloads, up from 51,000 downloads in October 2019—a laudable development! Equally gratifying is the submission rate. So far this year, 29 manuscripts have been submitted for the review process (as of early December 2020), which means that the number of submissions from last year has already been exceeded.

In addition to research articles that go through our standard double-blind peer-review, we also published other contributions this year. These include a number of insightful reviews and summary reports, a literature review by *Wenzelburger et al.* on the subject of *“Responsive policy-making in the German federal states? An attempt to systematize and develop a research program”* (1/2020), as well as an essay by *Klaus Ziemer* on *“Causes for the different development of democratic standards in the post-communist states of Europe”* (4/2020).

This issue (4/2020) contains five articles on the Special Section *Conceptualizing and Measuring Meanings and Understandings of Democracy*, which were published by the editors *Norma Osterberg-Kaufmann, Toralf Stark* and *Christoph Mohamad-Klotzbach* with a classifying contribution entitled *“Challenges in Conceptualizing and Measuring Meanings and Understandings of Democracy”*. This is the first *Special Section* to appear in our journal. We are very pleased that our focus formats are increasingly being used and are well received internationally. A thematic focus enables deeper examination of the research field, whereby the state of research can be presented in detail and developed further. In the coming year a Special Section will appear on *Environmentalism and Populism—an uneasy relation. Climate Movements in Comparative Perspective*, edited by *Aron Buzogány, Hans-Joachim Lauth* and *Christoph Mohamad-Klotzbach*. Two further Special Issues are currently in the planning stages: *Christiane Hubo, Simon Fink* and *Annette Elisabeth Töller* are preparing the Special Issue on *Party differences in environmental policy*. *Thomas Krumm* and *Marta Regalia* are also organizing a Special Issue on *Majority versus proportionality? Trajectories of electoral reform in the Mediterranean since the 1990s*.

The award for dissertations in the field of comparative area research was granted for the third time this year in honor of our colleague and co-editor Gero Erdmann. This year’s jury for the Gero Erdmann Prize, which comprises the work units in which Gero Erdmann played a key role, consisted of *Dr. Norma Osterberg-Kaufmann* (for the Democracy Research Working Group), *PD Dr. Andreas Ufen* (for the German Institute of Global and Area Studies) and *Prof. Dr. Matthijs Bogaards* (for the Journal of Comparative Politics). The jurors decided—from a wealth of nine submitted works—to award three dissertations.

The dissertation by *Theresia Smolka* on “Stress tests for the European Union. Change in the quality of democracy in the 27 member states between 2004 and 2012” received first place, worth €1000. *Markus Langenfeld* (dissertation on “Forest policy in Costa Rica and Chile. A comparative analysis of the tension between market and sustainability”) and *Franzisca Zanker* (dissertation on “Legitimacy in Peacebuilding: Rethinking Civil Society Involvement in Peace Negotiations”) share second place (each €500). We congratulate the award recipients on this success!^1^

Good news also comes from the editorial team. *Stephan Bröchler*, who i.a. oversees the reviews section, followed the call to the Berlin School of Law and Administration (HWR) to take over the professorship for political and administrative sciences. We would like to announce another exceptional appointment from the editorial team: at the recommendation of Federal Environment Minister Svenja Schulze, *Annette Elisabeth Töller* was appointed by the Federal Cabinet as one of seven experts in the Advisory Council for Environmental Issues (SRU) for the term of office from July 1, 2020 to mid-2024.^2^ We congratulate our two colleagues warmly!

Yet there is also sad news. We have been deeply saddened to hear of the passing of our esteemed colleague *Prof. Dr. Edeltraud Roller* (University of Mainz), who repeatedly worked as a constructive reviewer for our journal and still did so this year. Such commitment to our profession is admirable and we are very grateful to her for her support. We convey our deepest condolences to her relatives, friends and colleagues.

Another event is of great importance for the ZfVP as well as for German science in general. By signing Springer Nature and Projekt DEAL, the world’s most extensive open access transformation agreement, corresponding authors from a German university or research institution have been able to publish their articles *open access* in Springer Nature journals since January 1, 2020. The fees are covered by the DEAL agreement. The contract thus stands for more visibility, impact, efficiency, transparency and sustainability in the dissemination of the research results published in the ZfVP, which are now available free of charge for the benefit of the global scientific world.^3^ This is a great opportunity for our authors to communicate their research findings unhindered and on the basis of high quality standards.

Here we would like to highlight that the ZfVP is an excellent publication platform for comparative research and that we always welcome scientific contributions within this research context. You can send us your manuscript via the Editorial Manager: https://www.editorialmanager.com/zfvp/default.aspx. If you are interested in submitting reviews, comments, literature and conference reports, or if you would like to plan a special issue with us, please do not hesitate to contact us. For questions and suggestions, we can be reached at any time at zfvp@uni-wuerzburg.de. Further information on the ZfVP can be found at: https://www.springer.com/journal/12286.

We would like to thank all authors, reviewers and employees of the Springer VS publishing house for their cooperation during this challenging year. In particular, we would like to thank you for the numerous and informative reviews and reports that were written despite COVID-19’s adverse effects on science and on university operations.

In addition, we would like to thank *Christine Le Jeune* and *Jansen Harris* as well as *Anja Löbert* (https://www.textworks.eu/), who over the years have provided us with reliable and high-quality support on the proofreading and language correction of English manuscripts. We would also like to thank our student assistant *Pia Röhrer* for her support.

We very much appreciate all of your commitment and look forward to further cooperation in the coming year.

We wish you a good end to the year and the best of health!

Ein herausforderndes, aber dennoch ereignisreiches Jahr neigt sich dem Ende zu: Die weltweite COVID-19-Pandemie ist auch an der Zeitschrift für Vergleichende Politikwissenschaft (ZfVP) – insbesondere in der ersten Jahreshälfte – nicht spurlos vorübergegangen. Umso mehr freuen wir uns, dass auch im Jahr 2020 vier facettenreiche Ausgaben erschienen sind, die auf großes Interesse stoßen. Dies zeigt sich unter anderem an den Downloadzahlen, die stetig ansteigen. Während wir im Oktober 2019 51.000 Downloads verzeichneten, sind es im September 2020 bereits über 55.500 – eine erfreuliche Entwicklung! Auch die Einreichungsquote ist erfreulich. In diesem Jahr wurden bislang 29 Manuskripte für den Begutachtungsprozess eingereicht (Stand Anfang Dezember 2020) und damit ist bereits jetzt die Anzahl der Einreichungen aus dem letzten Jahr übertroffen.

Neben den klassischen Aufsätzen, die Standardgemäß unser double-blind peer-review durchlaufen, veröffentlichten wir auch in diesem Jahr andere Beiträge. Dazu zählen eine ganze Reihe aufschlussreicher Rezensionen und Sammelberichte, ein Literaturbericht von *Wenzelburger et al.* zum Thema *„Responsive Politikgestaltung in den deutschen Bundesländern? Versuch einer Systematisierung und Konzeption eines Forschungsprogramms“* (1/2020), sowie ein Essay von *Klaus Ziemer* zu *„Ursachen der unterschiedlichen Entwicklung demokratischer Standards in den postkommunistischen Staaten Europas“* (4/2020).

In diesem Heft (4/2020) befinden sich fünf Beiträge zur Special Section *Conceptualizing and Measuring Meanings and Understandings of Democracy*, die von den Herausgebenden *Norma Osterberg-Kaufmann, Toralf Stark* und *Christoph Mohamad-Klotzbach* durch einen einordnenden Beitrag mit dem Titel *„Challenges in Conceptualizing and Measuring Meanings and Understandings of Democracy“* eingeführt werden. Dies ist die erste Special Section, die in unserer Zeitschrift erscheint. Es freut uns sehr, dass unsere Schwerpunktformate vermehrt genutzt werden und diese international Anklang finden. Durch den Themenschwerpunkt wird eine tiefere Auseinandersetzung mit dem Forschungsfeld ermöglicht, und der Forschungsstand kann ausführlich dargestellt und weiterentwickelt werden. Im kommenden Jahr wird die Special Section zu *Environmentalism and Populism – an uneasy relation. Climate Movements in Comparative Perspective*, die von *Aron Buzogány, Hans-Joachim Lauth* und *Christoph Mohamad-Klotzbach* herausgegeben wird, erscheinen. Zudem sind zwei weitere Special Issues in Planung. *Christiane Hubo, Simon Fink* und *Annette Elisabeth Töller* bereiten das Sonderheft zu *Parteiendifferenz in der Umweltpolitik* vor. Unter der Leitung von *Thomas Krumm* und *Marta Regalia* wird zudem ein Special Issue zu *Majority versus proportionality? Trajectories of electoral reform in the Mediterranean since the 1990s* auf den Weg gebracht.

In ehrenvollem Andenken an unseren Kollegen und Mitherausgeber Gero Erdmann wurde in diesem Jahr zum dritten Mal der Förderpreis für Dissertationen aus dem Bereich der vergleichenden Area Forschung verliehen. Die diesjährige Jury des Gero Erdmann-Preises, die sich aus den Arbeitseinheiten bildet, an denen Gero Erdmann maßgeblich mitwirkte, setzte sich aus *Dr. Norma Osterberg-Kaufmann* (für den Arbeitskreis Demokratieforschung), *PD Dr. Andreas Ufen* (für das German Institute of Global and Area-Studies) und *Prof. Dr. Matthijs Bogaards* (für die Zeitschrift für Vergleichende Politikwissenschaft) zusammen. Die Juror*innen entschieden sich – aus der Fülle von neun eingereichten Arbeiten – drei Dissertationen zu prämieren.

Die Dissertation von *Theresia Smolka* zu „Belastungsproben für die Europäische Union. Veränderung der Demokratiequalität in den 27 Mitgliedstaaten zwischen 2004 und 2012“ wurde auf den ersten Platz gesetzt, der mit 1000 € dotiert ist. *Markus Langenfeld* (Dissertation zu „Waldpolitik in Costa Rica und Chile. Eine vergleichende Analyse des Spannungsfeldes zwischen Markt und Nachhaltigkeit“) und *Franzisca Zanker* (Dissertation zu „Legitimacy in Peacebuilding: Rethinking Civil Society Involvement in Peace Negotiations“) teilen sich den zweiten Platz (à 500 €). Wir gratulieren den Preisträger*innen zu diesem Erfolg!^4^

Auch im Rahmen der Redaktion gibt es erfreuliche Neuigkeiten. *Stephan Bröchler*, der u. a. die Rubrik der Rezensionen betreut, ist dem Ruf an die Hochschule für Recht und Verwaltung Berlin (HWR) gefolgt und übernimmt dort die Professur für Politik- und Verwaltungswissenschaften. Eine weitere exzeptionelle Berufung aus dem Redaktionskreis möchten wir gerne vermelden: *Annette Elisabeth Töller* wurde, auf Vorschlag der Bundesumweltministerin Svenja Schulze, vom Bundeskabinett als eine von sieben Expert*innen in den Sachverständigenrat für Umweltfragen (SRU) – für die Amtsperiode 1. Juli 2020 bis Mitte 2024 – berufen.^5^ Wir gratulieren unseren beiden Kolleg*innen sehr herzlich!

Dennoch gibt es auch eine traurige Nachricht. Mit Betroffenheit haben wir vom Tod unserer geschätzten Kollegin *Prof. Dr. Edeltraud Roller* (Universität Mainz) erfahren, die für unsere Zeitschrift immer wieder als konstruktive Gutachterin tätig war und dies auch noch in diesem Jahr war. Ein solcher Einsatz für unsere Profession ist bewundernswert und wir sind ihr für diese Unterstützung sehr dankbar. Unser Mitgefühl gilt den Angehörigen, Freunden und Kolleg*innen.

Ein anderes Ereignis hat eine gewichtige Bedeutung für die ZfVP sowie für die deutsche Wissenschaft im Allgemeinen. Durch die Unterzeichnung von Springer Nature und Projekt DEAL, des weltweit umfangreichsten Open Access-Transformationsvertrages, sind seit 1. Januar 2020 korrespondierende Autor*innen, die einer deutschen Universität oder Forschungseinrichtung angehören, berechtigt, ihre Artikel *Open Access* in Springer-Nature-Zeitschriften zu veröffentlichen, wobei die Gebühren unter die deutsche DEAL-Vereinbarung fallen. Der Vertrag steht somit für mehr Sichtbarkeit, Wirkung, Effizienz, Transparenz und Nachhaltigkeit bei der Verbreitung der in der ZfVP veröffentlichten Forschungsergebnisse, die nun zum Nutzen der globalen Wissenschaftswelt kostenfrei zur Verfügung stehen.^6^ Dies ist eine große Chance für unsere Autor*innen ihre Forschungserkenntnisse ungehindert und auf Basis hoher Qualitätsstandards zu kommunizieren.

An dieser Stelle wollen wir darauf hinweisen, dass die ZfVP eine hervorragende Publikationsplattform für komparative Forschung darstellt und wir wissenschaftliche Beiträge aus diesem Forschungskontext stets begrüßen. Ihr Manuskript können Sie uns über den Editorial Manager zukommen lassen: https://www.editorialmanager.com/zfvp/default.aspx. Falls Sie Interesse haben Rezensionen, Kommentare, Literatur- sowie Tagungsberichte einzureichen, oder ein Sonderheft mit uns planen wollen, dann nehmen Sie gerne Kontakt mit uns auf. Für Fragen und Anregungen sind wir jederzeit unter zfvp@uni-wuerzburg.de erreichbar. Weitere Informationen zur ZfVP finden Sie unter: https://www.springer.com/journal/12286.

Allen Autor*innen, Gutachter*innen und Mitarbeiter*innen des Verlags Springer VS gilt unser Dank für die Zusammenarbeit in diesem herausfordernden Jahr. Insbesondere möchten wir uns für die Anfertigung der zahlreichen sowie aufschlussreichen Rezensionen und Gutachten, die trotz der Corona bedingten Beeinträchtigungen des Wissenschafts- und Hochschulbetriebs verfasst wurden, herzlich bedanken.

Zudem gilt unser großer Dank *Christine Le Jeune* und *Jansen Harris* sowie *Anja Löbert* (https://www.textworks.eu/), die uns bei der Sprachkorrektur der englischen Manuskripte im Laufe der Jahre zuverlässig und qualitativ hochwertig unterstützt haben. Unserer studentischen Hilfskraft *Pia Röhrer* danken wir ebenfalls für die Unterstützung.

All ihr Engagement schätzen wir sehr und freuen uns auf die Kooperationen im nächsten Jahr.

Wir wünschen Ihnen einen guten Jahresabschluss in bester Gesundheit!

## Reviewer 2019/2020

Pamela Abbott, Hartmut Aden, Michael Alvarez, Gal Ariely, Christiane Barnickel, Youngho Cho, Lasse Cronqvist, Judith Enders, Katalin Fabian, Eszter Farkas, Martin Florack, Jean-Paul Gagnon, Brigitte Geißel, Manuela Glaab, Tobias Haas, Mark Heckmann, Lea Heyne, Oliver Hidalgo, Ekrem Karococ, Pascal König, András Körösényi, Matthias Lemke, Natasha Lindstaedt, Robert Mattes, Marcus Maurer, Anja Mays, Katja Müller, Abel Reiberg, Michael Robbins, Pedro L. Rodríguez, Robert J. Rohrschneider, Edeltraud Roller, Hannes Schammann, Andreas Schedler, Harald Schoen, Wolf Jürgen Schünemann, Doh Chull Shin, Markus B. Siewert, Alexander Thumfart, Luca Tomini, Aiko Wagner, Michael Wahman, Alexander Weiß

## Footnotes

1 See the laudations of the jury in issue 3/2020 of the ZfVP.

2 See also https://www.bmu.de/pressemitteilung/bundesumweltministerin-svenja-schulze-beruft-neuen-sachverstaendigenrat-fuer-umweltfragen/.

3 See also https://www.hrk.de/presse/pressemitteilungen/pressemitteilung/meldung/springer-nature-und-projekt-deal-unterzeichnen-weltweit-umfangreichsten-open-access-transformationsv/.

4 Siehe hierzu die Laudationes der Jury in Ausgabe 3/2020 der ZfVP.

5 Siehe hierzu https://www.bmu.de/pressemitteilung/bundesumweltministerin-svenja-schulze-beruft-neuen-sachverstaendigenrat-fuer-umweltfragen/.

6 Siehe hierzu https://www.hrk.de/presse/pressemitteilungen/pressemitteilung/meldung/springer-nature-und-projekt-deal-unterzeichnen-weltweit-umfangreichsten-open-access-transformationsv/.

